# Diagnostic Value of Radioisotope Cisternography Using ^111^In-DTPA in a Patient with Rhinorrhea and Purulent Meningitis

**DOI:** 10.3390/medicina58060714

**Published:** 2022-05-26

**Authors:** Stefan Porubcin, Alena Rovnakova, Ondrej Zahornacky, Pavol Jarcuska

**Affiliations:** 1 The Department of Infectious Diseases and Travel Medicine, Louis Pasteur University Hospital, Rastislavova 43, 04011 Kosice, Slovakia; stefan.porubcin@gmail.com (S.P.); alenarovnakova@gmail.com (A.R.); 2 Faculty of Medicine, Pavol Jozef Safarik University in Kosice, Trieda SNP No. 1, 04011 Kosice, Slovakia

**Keywords:** meningitis, rhinorrhea, cisternography

## Abstract

Cerebrospinal fluid (CSF) leakage is a rare condition. Prompt diagnosis and early treatment of CSF leakage minimizes the risk of severe complications such as bacterial meningitis. Different diagnostic modalities are used to detect the site of CSF leakage but often with unreliable results. The literature offers limited evidence-based guidance on the diagnostic approach for rhinorrhea. Correct localization of the defect is the mainstay for successful surgical treatment. Herein, we describe a case of recurrent meningitis due to cranio-nasal fistula and rhinorrhea successfully localized with radioisotope cisternography (RIC). We provide a detailed and practical overview of the RIC procedure and compare different imaging modalities used to detect the site of CSF leakage.

## 1. Introduction

Cerebrospinal fluid rhinorrhea is the result of an abnormal communication between the subarachnoid space and the sinonasal tract [1]. CSF leaks in adults generally occur following skull-based trauma or neurosurgical procedure and can cause serious complications, e.g., meningitis (reported to range from 10% to 37% during conservative management), unless treated properly [2]. The correct demonstration of the leak localization is of utmost importance while planning a surgical intervention to prevent future episodes of recurrent meningitis. In patients with a suspected CSF fistula, it is difficult to detect the leak localization with routine cranial high-resolution CT (HRCT) or MRI. Contrast-enhanced magnetic resonance cisternography (CE-MRC) is difficult to implement because intrathecal administration of gadolinium-based contrast agents can be associated with serious neurotoxic complications [3]. We describe an interesting case report of a patient with recurrent bacterial meningitis and the use of radioisotope cisternography (RIC) with ^111^In-DTPA radioisotope and single-photon emission computed tomography (SPECT) as a method for definitive anatomical localization of cranio-nasal fistula.

## 2. Case Report

A 23-year-old man with a history of cranio-cerebral trauma was admitted to the Department of Infectious Diseases and Travel Medicine for torpid headache, nausea, vomiting, and altered mental status. His symptoms started one day before. He had a history of a previous episode of purulent meningitis (7 years earlier).

During the initial evaluation, his vital signs were as follows: blood pressure 150/90 mm Hg, pulse was 80 and regular, oxygen saturation 97% on room air, temperature was 38 °C, and respiratory rate 30. Heart, abdomen, and legs were normal. No skin petechiae were observed.

On neurologic examination, he was somnolent, disorientated, had nuchal rigidity, and positive Lasegue and Kernig sign. No cranial nerve abnormalities were present. Lumbar puncture (LP) was performed during the first hour of admission. The macroscopic appearance of the CSF was purulent. White blood cell count: >5000 cells/µL (>99% polymorphonuclears), glucose level: 1.75 mmol/L, chloride level: 120 mmol/L, protein level: 3.86 g/L, lactate level: 10.1 mmol/L (reference ranges in Table 1). The diagnosis of bacterial meningitis was established.

The patient was admitted to the ICU and was started on third-generation cephalosporine (cefotaxime 3 g q6h). He was administered dexamethasone, mannitol (osmotic diuretic), intravenous fluids, and vitamin C as well. Chest X-ray was negative. Computed tomography (CT) of the brain and paranasal sinuses showed small irregularity in the cribriform plate, over the frontal and ethmoidal sinuses, with a small pathological collection inside right frontal, ethmoidal, and maxillary sinus. No brain edema, hydrocephalus, or abscess formation was observed. Despite being on sedative medication, the patient was restless and agitated, and therefore some CT images were blurry and could not be evaluated accurately. Blood and urine cultures were negative. Nasal and throat swabs were positive for Staphylococcus epidermidis. We also conducted a lumbar puncture to gain CSF for microbiological examination. There was a positivity of latex agglutination for Streptococcus pneumoniae in CSF and also positive CSF culture for the same pathogen with good sensitivity to all tested antibiotics. All relevant laboratory results were measured in whole blood/plasma and are summarized in Table 1.

One day after the initiation of antibiotic treatment, the neurological status improved, and the patient was responsive and able to communicate. There was defervescence of fever. Subsequently, the patient started to complain of rhinorrhea. He told the treating physician that he had had intermittent rhinorrhea for years. Neurosurgical consultation suggested RIC as a method for visualization of assumed cranio-nasal communication.

**Figure 1:**
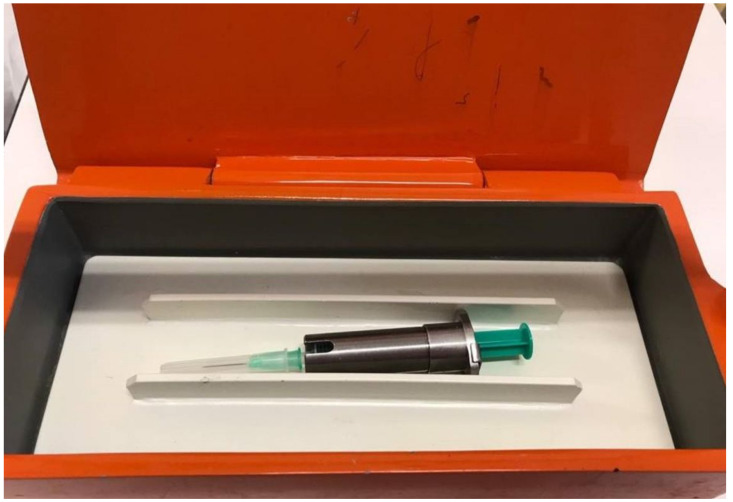
Shielded syringe with radioisotope (2 mL of ^111^In-DTPA).

**Figure 2:**
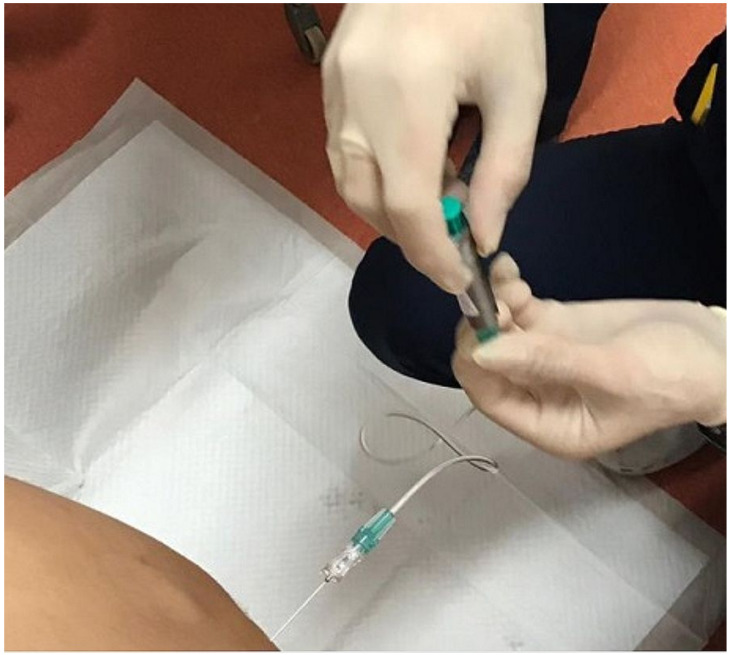
Application of the radioisotope via LP needle with additional tubing.

At the end of the antibiotic treatment (14th day), in cooperation with the Institute of Nuclear and Molecular Medicine, we decided to perform RIC. The radioisotope ^111^In-DTPA with 42 MBq (2 mL) was introduced into the spinal subarachnoid space via the LP. For the radioisotope to be fully introduced and dispersed into the subarachnoid space, without leaving any residues inside the LP needle and to counteract the backwash through the LP needle, we had to institute additional 3 mL of normo-saline. The whole procedure had to be performed with the help of additional tubing, otherwise the radioisotope would leak out after we disconnected the syringe from the LP needle (Figure 1 and Figure 2). Afterwards, we placed two pledgets inside the nasal cavity for the purpose of subsequent lateralization of rhinorrhea. This was then followed by images taken at specified time intervals (6 and 24 h post-application) to assess the distribution of the activity as it expanded cephalad from its lumbar subarachnoid location.

**Figure 3:**
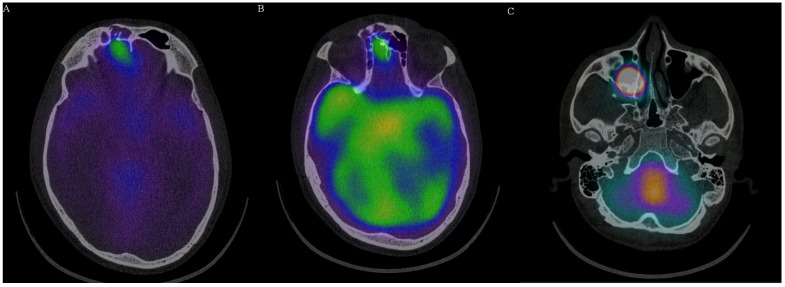
SPECT scanning: (**A**) axial image of increased radioisotope activity over the right frontal sinus with bone defect; (**B**) axial image of increased radioisotope activity over the anterior part cribriform plate with bone defect; (**C**) radioisotope activity in the right maxillary sinus. (Images provided with the courtesy of the Institute on Nuclear and Molecular Medicine, Kosice.).

**Figure 4:**
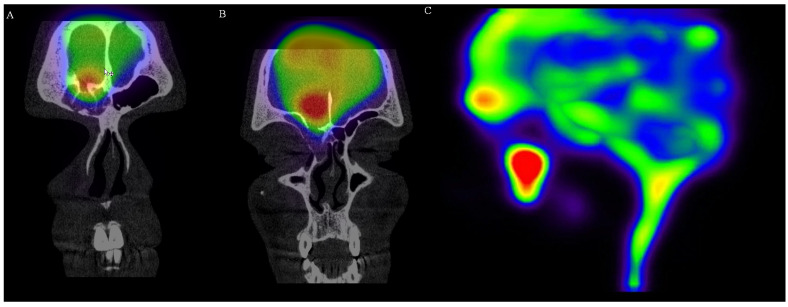
SPECT scanning: (**A**) coronal image of increased radioisotope activity over the right frontal sinus with bone defect; (**B**) coronal image of increased radioisotope activity over the anterior part cribriform plate with bone defect; (**C**) SPECT scan showing increased activity in the frontal area and the cumulation of radioisotope in the right maxillary sinus. (Images provided with the courtesy of the Institute on Nuclear and Molecular Medicine, Kosice.).

Early scanning (5–6 h) showed symmetrical activity in the ventricular system. There was no activity in the liver, which confirmed correct application of the radioisotope. Sagittal images showed slight atypical activity caudally of the cribriform plate. Except physiological distribution, delayed scanning (>24 h) revealed the focus of an increased activity over the cribriform plate and extremely intense activity extracranially inside right paranasal cavity. Planar images of the abdominal area showed activity in the spinal canal and swallowed activity in the stomach and intestines, which also confirmed liquorrhea. Single photon emission computed tomography (SPECT) scanning clearly confirmed activity over the internal lamina of the frontal bone, as well as over the anterior part of cribriform plate (two defects). There was a posttraumatic discontinuity of the skull base (communication between cranium and frontal sinus 6.3 mm in diameter) and a second defect in the anterior part of the cribriform plate. The first signs of the isotope leakage were evident at 6 h post-application. After 24 h, the activity was evident in communicating fistula and inside the right maxillary sinus (Figure 3 and Figure 4, Video S1). Finally, we removed the nasal pledgets and measured the radioisotope activity. There was a distinct side difference. The right-sided pledget had a measured activity of 159 kBq, and the left one, 5.0 kBq. The activity on the right side was more than 30 times higher.

The patient was then transferred to the Department of Neurosurgery. Following a successful localization of the fistula, the surgeon performed a reconstruction of the anterior cranial fossa floor through right frontal craniotomy. The patient was discharged 12 days after the surgery and has had no recurrence of rhinorrhea since.

## 3. Discussion

Bacterial meningitis in patients with CSF leakage can be classified as community acquired, in the case of anatomic defects or contiguous spread of infection, or nosocomial meningitis after surgery for trauma. In patients with rhinorrhea, the diagnosis should first be confirmed by measurement of beta-2-transferrin levels. This protein is specific for CSF, and a small amount of rhinal discharge (0.4 mL) is adequate for the evaluation. During the evaluation of our patient, this laboratory measurement was not conducted because of the clinical picture with suggestive history of recurrent meningitis and actual large volume rhinorrhea. The most common causative pathogens are *S. pneumoniae* and *H. influenzae*, and the outcome of these episodes is generally favorable [4]. We have confirmed Streptococcus pneumoniae by culture and latex agglutination. The patient was not vaccinated, which could have prevented meningitis. However, this is not universal. In the last decade, some patients at our department, who have been diagnosed with recurrent meningitis due to rhinorrhea and had been vaccinated, had an etiological pneumococcal serotype, which is not included in the pneumococcal polysaccharide vaccine. Our patient had pneumococcal serotype 23B. Similarly, Van der Linden observed significant increase in the prevalence of pneumococcal serotypes 15A and 23B between 2007 and 2014, not included in the vaccine [5]. The particular strain was sensitive to all tested antibiotics.

Many imaging methods, including cranial CT, MRI, MR cisternography (MRC), contrast-enhanced MRI (CE-MRI), or RIC, are used to detect the location of CSF leakage, all with their advantages and limitations. HRCT can indicate the leak localization by detecting the bone defect. However, CSF leakage may not be present at the congenital or traumatic defect localization [6]. In our case, the initial CT imaging was not able to detect the localization of the cranio-nasal communication, nor to confirm the side of the pathology. MRI and MRC cohort studies show sensitivity and specificity of 90% and 77%, respectively [7]. Avoidance of intrathecal injection of contrast is a key benefit of MR cisternography. T2-weighted imaging can be used to detect the presence of CSF in the sinonasal cavity without the invasiveness of contrast injection. However, both CT and MR studies have limited ability in detecting intermittent rhinorrhea [8]. In our patient, we omitted MRI imaging because of immediate availability of RIC. CE-MRI with intrathecal administration of gadolinium is not approved in many countries, and therefore is not an option for most clinicians [9].

RIC is an invasive study, but LP as a method for instituting the radioactive tracer is a simple and safe procedure. This method is mainly used in cases where there is still diagnostic uncertainty. The cephalad ascent of the radioisotope is not affected by activity or by posture (horizontal or upright) after the injection; therefore, the patient has no limitation in his activities [10]. Different tracers, including radioactive ^131^I, diethylenetriamine pentaacetic acid (DTPA), ^111^In-DTPA, ^99^Tc human serum albumin, and ^99^Tc pertechnetate can be used. ^99^Tc-DTPA has been suggested as a better brain scanning agent than ^99^Tc pertechnetate because its renal clearance from the blood stream is more rapid, presumably resulting in better lesion-to-background ratio at earlier times after injection, and there is no interference from choroid plexus and salivary gland concentration. Clinically, marked reduction in the time delay between injection and imaging to 1 h or less has been advocated because such early studies with DTPA were felt to be at least as reliable as pertechnetate images 2 or more hours after injection [11]. At our institution, we prefer ^111^In over ^99^Tc because of longer half-life (67 h vs. 6 h), which is advantageous in visualization of intermittent leaks [12]. Moreover, as opposed to some other radioisotopes, the incidence of aseptic meningitis with ^111^In has been rare [13]. Radiation dose exposure of ^111^In RIC is 5 mSv at most, while radiation from contrast enhanced CT cisternography may expose the patient to up to eight times more [14]. ^99^Tc-DTPA has been suggested as a better brain scanning agent than ^99^Tc pertechnetate because its renal clearance from the blood stream is more rapid, presumably resulting in better lesion-to-background ratio at earlier times after injection, and there is no interference from choroid plexus and salivary gland concentration. Clinically, marked reduction in the time delay between injection and imaging to 1 h or less has been advocated because such early studies with DTPA were felt to be at least as reliable as pertechnetate images 2 or more hours after injection [15].

Localization of the leak to the right or left nasal cavity may be sometimes difficult because of the tendency of the fluid to cross sides and flow from both nostrils. In a study of four patients who underwent RIC, as well as MRI and/or CT for suspected CSF leaks, Thomas et al. found that RIC accurately detected and localized the leaks in all patients [15]. The tracer reaches the skull base in 6 h and is present over the cerebral convexities in 24 h. Cotton pledgets that are placed in the nose pick up the isotope, and the difference in activity is helpful in lateralization of CFS leakage if other studies fail. This was demonstrated in our patient as well. There was a clear side difference of radioisotope accumulation. We had omitted unnecessary image acquisition at 0, 1, and 3 h, which is mainly helpful in spinal leaks [16].

As with all imaging methods, there are some issues with RIC that need to be considered. After an LP, commonly a small amount of CSF may leak from the puncture site. This is called a backwash phenomenon. If the amount of the backwash were large, stronger lumbar epidural radioactivity might be seen in the cisternographic images and could lead to early appearance of radioactivity in the urinary bladder [17]. Multiple puncture attempts due to difficult LPs have been blamed by some for this phenomenon [18]. It is also an invasive study and therefore carries the potential risks inherent to any other lumbar puncture with intrathecal injection. In addition, measuring pledget activity cannot guarantee that the CSF is from a rhinologic leak and not an otologic leak that has traveled down the Eustachian tube into the nasopharynx. However, SPECT scanning can reveal a pathological site in most cases.

In the spectrum of neurodiagnostic tests in evaluation of the patients with spontaneous CSF leaks, RIC has its own particular indication and is not necessarily a substitute or competitor to other imaging techniques. 

## 4. Conclusions

For an infectious disease specialist or neurologist, recurrent meningitis should be a sign of possible CSF leakage and should lead to prompt imaging to determine the site of skull-based CSF leak. Although vaccination prevents meningitis, some cases may be caused by streptococcal serotypes not included in the vaccine, as documented in our case. There are many different imaging modalities, each with its advantages and caveats. This case report demonstrates the significance and the practical approach to RIC with SPECT in cases of rhinorrhea, being a method for definitive visualization of cranio-nasal communication. ^111^In-DTPA is the preferred radioisotope of choice for its longer half-life and the ability to detect intermittent leaks. Timely detection of anatomical defect thus allows for targeted surgical treatment.

## Figures and Tables

**Table 1:** Laboratory values.

Variable	Reference Range, Adults	On Admission	Follow-Up *
Hematocrit (%)	36–46	47	41
Hemoglobin (g/dL)	12–16	15.9	14.1
White cell count (×10^9^/L)	4.0–10	**26**	13
Differential count (×10^9^/L)			
Neutrophils	1.4–6.5	**24.12**	11.39
Lymphocytes	1.5–4.0	0.67	0.8
Monocytes	0.25–0.6	1.1	1.02
Eosinophils	0.05–0.25	0.03	0.0
Platelet count (×10^9^/L)	150–400	238	274
Prothrombin–time international normalized ratio	0.85–1.15	1.19	1.07
Fibrinogen (g/L)	1.8–3.5	**10.4**	**1.9**
C-reactive protein (mg/L)	<5	**391**	**8.45**
Urea (mmol/L)	2.8–7.2	6.86	5.37
Creatinine (mmol/L)	49–90	91.6	82
Aspartate aminotransferase (µkat/L)	0.05–0.6	0.47	0.27
Alanine aminotransferase (µkat/L)	0.05–0.6	0.26	0.36
Gamma glutamyl transpeptidase (µkat/L)	0.05–0.63	0.30	0.28
Alkaline phosphatase (µkat/L)	0.5–2	1.32	1.09
Procalcitonin (µg/L)	<0.5	**25.2**	0.1
Lactate (mmol/L)	0.5–2.2	**2.6**	1.7
Urine culture	-	negative	-
Blood culture	-	negative	-
CSF culture	-	*Streptococcus pneumoniae*	negative
CSF Latex agglutination *Streptococcus pneumoniae*	-	**positive**	negative
CSF neutrophils (/mm^3^)	0–10	>**5000**	13
CSF protein level (g/L)	<0.5	**3.86**	0.56
CSF glucose level (mmol/L)	2.5–4.4	**1.75**	3.2
CSF chloride level (mmol/L)	116–127	120	122
CSF lactate (mmol/L)	1.1–2.4	**10.1**	1.68

Font in bold: pathological value. * Follow-up laboratory results were taken 4 days after the initial evaluation; follow-up CSF examination was repeated after 13 days.

## Data Availability

Not applicable.

## Supplementary Materials

The following supporting information can be downloaded at https://www.mdpi.com/article/10.3390/medicina58060714/s1. Video S1: 3D visualization of cranio-nasal communication (video provided with the courtesy of the Institute on Nuclear and Molecular Medicine).
